# Diet, Sleep, and Mental Health: Insights from the UK Biobank Study

**DOI:** 10.3390/nu13082573

**Published:** 2021-07-27

**Authors:** Piril Hepsomali, John A. Groeger

**Affiliations:** 1 Department of Psychology, University of Roehampton, London SW15 4JD, UK; 2 Unilever R&D, Colworth Science Park, Bedford MK44 1LQ, UK; 3 Department of Psychology, School of Social Sciences, Nottingham Trent University, Nottingham NG1 4BU, UK; john.groeger@ntu.ac.uk

**Keywords:** diet, sleep quality, mental health, anxiety, depression

## Abstract

Diet has long been the focus of attention as a leading risk factor for non-communicable diseases. As such, a better understanding of it is crucial to establish priorities for dietary guidelines and to inform, design, and implement strategies for preventing, helping manage, and stopping the progression of sleep and mental health-related symptoms/disorders. The aim of the current study is to conduct the largest investigation of diet, sleep, and mental health to date by utilizing the UK Biobank (UKB) dataset to identify the associations between diet and (i) sleep quality/health, and (ii) mental health symptomatology. This cross-sectional population-based study involved 502,494 middle-aged adults. UKB food frequency, sleep, and psychological factors and mental health questionnaires at baseline were used. Scores were also calculated for healthy diet, healthy sleep, mental health symptomatology, partial fibre intake, and milk intake. We observed positive associations with healthy diet and sleep and mental health, especially benefits of high intakes of vegetable, fruit, fish, water, and fibre. However, processed meat and milk intake were adversely associated with sleep and mental health. These findings make clear that there are health and wellbeing benefits and drawbacks of different diets, but do not, at this stage, demonstrate the clear causal relationships, which would support dietary interventions that might play a role in the treatment and also self-management of sleep and mental health disorders/symptoms. Further research is required to understand mechanisms of actions of which diet acts on to modulate sleep and mental health, while taking comorbidity of sleep and mental health disorders/symptoms into consideration.

## 1. Introduction

Studies from countries across the world have reported a prevalence of sleep problems ranging from 1.6% to 56.0% [1,2,3]. Similarly, common mental disorders are highly ubiquitous; specifically, depressive and anxiety disorders affect more than 300 million and 250 million people, respectively [4]. It is also important to note that anxiety and depressive disorders are highly comorbid [5] and that there is a bi-directional relationship between sleep and mental health. For instance, sleep disturbances have been observed in individuals with anxiety [6] and depression [7]. Additionally, having a sleep disturbance may predict the development of an anxiety disorder [8] and depression [9].

The associated social, economic, and health burdens of sleep and mental health disorders cost governments trillions of dollars each year [10,11] due to their high prevalence. Additionally, subclinical sleep, depression, and anxiety symptoms are highly prevalent across population; hence, they may add even further personal and economic burden on a population scale. Although standard treatments such as pharmacotherapy and psychotherapy are considered as first-line treatments, due to their modest to moderate efficacy even in the clinical populations [12,13,14] and the size of the public health impact, new primary and/or complementary methods to improve and prevent symptoms of poor sleep and mental health across the population are urgently needed.

A relatively new research field, nutritional psychiatry [15,16] aims to investigate the effect of dietary patterns, diet quality, and dietary components on mental and sleep health, providing promising evidence for addressing this large disease burden. Research to date has shown that various nutrients, nutritional deficiencies or abundancies, food items, diet quality, and diet type may affect sleep health and mental health [17,18,19]. It is believed that dietary factors affect sleep via (a) circulating intestinal hormones (e.g., insulin, CCK, ghrelin, PYY), (ii) by stimulating the synthesis of serotonin and melatonin, (iii) acting on GABAergic or serotonergic neurons, or (iv) via other unidentified mechanisms [20]. On the other hand, research investigating the dietary mechanisms of actions that diet could potentiate mental health changes identifies (i) inflammation, (ii) oxidative stress, and (iii) neuroplasticity, with the gut microbiome as a key mediating pathway for each of these processes [21,22]. It is important to note that the microbiota–gut–brain axis has been identified as the primary nutrient-mediated pathway that is involved in the regulation of both sleep health and mental health [23,24]. 

A healthy/high-quality diet is predominantly plant-forward, and is characterised by the high intake of vegetables, fruits, wholegrains, and fish, with limited intake of other animal-based foods. In contrast, unhealthy/low-quality diets (e.g., increased consumption of Western-type foods) are high in fat and sugar [21,25,26]. Healthy/high-quality diets are generally high in fibre [21,27], and both fibre intake [28,29] and Mediterranean-style healthy diets [30,31] are known to be associated with beneficial changes in the gut microbiome composition. It has also been shown that elements commonly found in unhealthy/low-quality (Western-style) diets, such as saturated fatty acids, were associated with negative alterations in the microbiota [32]. Given the impact of gut-microbiome on brain and behaviour [23,33], modulating the gut-microbiome by adhering to a healthy diet may be a plausible strategy for developing a potentially useful and efficacious approach to sleep and mental condition and/or symptom treatment and even prevention. 

Research on the associations between healthy/high-quality dietary patterns and sleep is limited to the positive associations between Mediterranean diet and sleep quality [34]. On the other hand, although heterogenous, and mostly based on individual studies in small study populations, experimental research to date showed beneficial effects of: large quantities of carbohydrates and fats; proteins (amino acid tryptophan); group B vitamins and magnesium supplementation; supplements and foods containing tryptophan, melatonin, and phytonutrients (e.g., cherries, kiwifruit, milk) on various sleep outcomes (see reviews [20,35,36]). Larger epidemiology-based studies found associations between higher fat intake and sleep disorders, adherence to Mediterranean diet and fewer insomnia symptoms in women, and conflicting evidence regarding carbohydrate intake and sleep quality (for a review, see [36]). Since people do not consume single nutrients or supplements in isolation, nutritional research is gradually shifting towards food group and dietary pattern analysis [37]. However, as there is only very limited evidence showing associations between diet quality, food groups, and sleep, it is crucial to uncover these relationships in a large cohort study as a first step. 

On the other hand, unlike sleep, there is a substantial amount of evidence showing the benefits of healthy/high-quality diet on mental health. Meta-analytic and systematic data showed that healthy dietary patterns (higher intakes of fruit, vegetables, fish, and wholegrains) are negatively associated with increased risk of depression, but not anxiety [25,38,39,40]. Epidemiology-based studies have also indicated that unhealthy dietary patterns (higher intakes of products high in fat and sugar) are positively associated with risk of depression and anxiety [41,42]. However, well-controlled randomised-controlled trials (RCTs) show mixed results. Some studies provide evidence of improvement of major depressive episodes severity [43,44] and alleviation of depression symptoms [45] with adherence to a Mediterranean-style diet. Other studies, however, fail to show a positive effect of multi-nutrient supplementation (omega-3 fatty acids, selenium, folic acid, vitamin D3, and calcium) on major depressive episode severity in overweight or obese adults with subsyndromal depressive symptoms [46]. Additionally, studies have examined the effects of individual or multiple food groups in relation to mental health and showed positive effects of fish [47], fruits and vegetables [48,49], and fibre [50]. Although the amount of evidence showing positive associations between healthy dietary patterns and mental health is not negligible, the nature of these associations is not well characterised. First of all, in most of the previous studies, depression and anxiety have been assessed individually, without taking into account the comorbidity of these clinical conditions and/or subclinical symptoms. However, in order to (i) fully understand the applicability of and (ii) develop science-based diet improvement therapies as a primary or complementary method for improving the population’s general mental health and wellbeing, it is essential to understand the role of diet on overall mental health and wellbeing. Secondly, although there is some evidence regarding the contribution of specific food groups to better mental health, due to the sample size and heterogeneity of these studies, there is a need to establish whether better overall mental health is attributed to some or all components of healthy/good-quality diets via a large sample sized study. Thirdly, as sleep problems can promote or sustain mental health challenges, and vice versa, the role of diet in relation to (i) sleep per se and (ii) mental health per se, should also, ideally, be determined simultaneously. 

This study seeks to provide a better understanding of the associations between diet quality and dietary patterns of food group consumption and sleep, mental health, as well as their interdependence. Such an understanding could, we believe, help to establish priorities for dietary guidelines, and to inform, design, and implement strategies for preventing, stopping the progression of, and helping with the management of (i) sleep and mental health-related symptoms/disorders and/or (ii) those diet-related diseases, which may have a negative impact on sleep and mental health. Therefore, in the current study, we aimed to conduct the largest investigation of diet and sleep and mental health to date by systematically examining the UK Biobank (UKB) dataset, to identify the associations of sleep quality/health (based on chronotype, duration, insomnia, snoring, and excessive daytime sleepiness measures) and mental health symptomatology (based on total number of mental health-related complaints) with (i) the intake of food items and food groups including vegetables, fruits, processed and unprocessed meat, fish, tea, coffee, water, milk, as well as fibre and (ii) diet quality/healthy diet score.

## 2. Materials and Methods

This retrospective cohort study is based on data from the UKB study [51] that received approval from the National Information Governance Board for Health and Social Care and the National Health Service Northwest Multi-Centre Research Ethics Committee (Ref: 11/NW/0382). All participants provided informed consent through electronic signature at baseline assessment.

### 2.1. Study Population

Study design and methods of UKB have been reported in detail previously [51]. In brief, adults aged 40–69 years who were registered with the United Kingdom National Health Service and living within 25 miles of an assessment centre were invited to participate. Approximately nine million invitations were sent out, and data were collected from more than 500,000 eligible and consenting participants between 2006 and 2010. At a baseline visit, after providing written informed consent, participants completed a touch screen questionnaire that assessed various sociodemographic, lifestyle, and health behaviour variables. 

### 2.2. Assessment of Diet and Calculation of Diet-Related Scores

In order to assess diet, we used UKB Food Frequency Questionnaire scores at baseline. The questions used in this manuscript are those that asked about the frequency of consumption of cooked vegetables, salad/raw vegetables, fresh fruit, dried fruit, oily fish, non-oily fish, processed meat, poultry, beef, lamb/mutton, pork, bread, cereal, tea, coffee, and water. More detail about the questions, and possible responses, is given in the Supplementary (Sup.) Methods.

We also created various new data fields including (1) total vegetable intake, (2) total fruit intake, (3) total unprocessed red meat intake, (5) total fish intake, (6) healthy diet score, (7) partial fibre score, and (8) milk intake. In order to calculate total vegetable and fruit intake, we summed UKB cooked and UKB salad/raw vegetable intake and UKB fresh and UKB dried fruit intake, respectively. We also added UKB beef, UKB lamb/mutton, and UKB pork intake scores and UKB oily and UKB non-oily fish intake scores to create total unprocessed red meat intake and total fish intake scores, respectively. Finally, we utilised healthy diet score calculations to create composite healthy diet scores [52], partial fibre score calculations to create fibre scores [53], and milk intake calculations to create milk intake estimations [54] (please see Sup. Methods for a brief description of calculations). For both partial fibre score and milk intake, we created low, low/medium, medium, high/medium, and high intake groups based on quintile splits. 

### 2.3. Assessment of Sleep and Calculation of Healthy Sleep Score

In order to assess sleep, we used UKB’s touchscreen questionnaire on sleep at baseline. Data fields of interest were sleep duration, chronotype, sleeplessness/insomnia, snoring, and daytime dozing/sleeping. More detail about the questions, and possible responses, is given in the Sup. Methods.

In order to calculate a composite sleep score, we utilised healthy sleep score estimations (questions included in score calculations were equally weighted) based on a previous UKB publication [55], where higher scores represent healthier sleep patterns (please see Sup. Methods for a brief description of calculations). 

### 2.4. Assessment of Mental Health and Calculation of Composite Mental Health Score

For mental health assessment, we utilised UKB’s touchscreen questionnaire on psychological factors and mental health at baseline. Data fields of interest were mood swings, miserableness, irritability, sensitivity/hurt feelings, fed-up feelings, nervous feelings, worrier/anxious feelings, tense/highly strung, worry too long after embarrassment, suffer from nerves, loneliness/isolation, guilty feelings, and risk taking. More detail about the questions, and possible responses, is given in the Sup. Methods.

We calculated the total mental health complaints reported by adding up participant’s answers to the 13 UKB mental health questions described above, where higher numbers represent more mental health-related symptomatology. 

### 2.5. Statistical Analyses

All analyses were performed in IBM SPSS Statistics 26.0.0.0. Questionnaire response options, ‘do not know’ or ‘prefer not to answer’, were treated as missing values. Separate univariate ANOVA’s and ANCOVA’s (adjusted for age, sex, BMI) were used to examine the associations between dietary scores that were estimated from UKB data (healthy diet score, milk intake, and partial fibre intake) and healthy sleep score as well as mental health symptomatology. Bonferroni post hoc tests were used where appropriate. Separate unadjusted and adjusted (for age, sex, BMI, healthy sleep score, and mental health symptomatology) linear regressions were performed to examine the associations of food groups (vegetable, fruit, fish, unprocessed red meat, processed meat) and food items (water, tea, coffee) with (i) healthy sleep score and (ii) mental health symptomatology. None of the estimated dietary scores (i.e., healthy diet score, partial fibre intake, and milk intake) were included in any of the regression models.

## 3. Results

As seen in the Supplementary Data Table S1, of 502,494 participants, only 7.9% of the participants adhered to a healthy diet (healthy diet score 4 and above). Participants with higher healthy diet scores had lower measures of BMI and were more likely to be women. Additionally, participants with higher fibre and milk intake were more likely to be women, older, and more affluent (see Supplementary Data, Tables S2 and S3). In terms of sleep, Table S4 shows baseline characteristics of participants according to healthy sleep score. Participants with healthy sleep scores had lower measures of BMI, were more likely to be women, younger, and more affluent. Finally, characteristics of mental health symptomatology are reported in Supplementary Data Table S5. Cronbach’s alpha was 0.81 for this measure, and more detailed analysis concerning reliability can be seen in Supplementary Data Tables S6 and S7.

### 3.1. Diet and Sleep

#### 3.1.1. Associations between Diet Quality on Sleep

**Figure 1:**
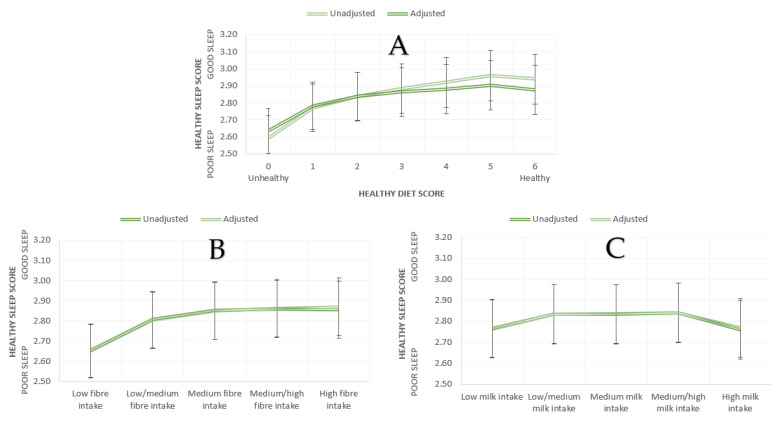
Mean healthy sleep scores according to (**A**) healthy diet scores (**B**) fibre intake, (**C**) milk intake (bars represent 95% CI).

After excluding diet score of seven (i.e., highest healthy diet score) due to low number of participants (n = 1) from the analyses, we conducted univariate ANOVA and ANCOVA (adjusted for age, sex, BMI) with healthy diet score as an independent variable and healthy sleep score as a dependent variable. We observed a significant main effect of healthy diet score on healthy sleep score in the unadjusted (F(6, 502485) = 592.21, *p* < 0.001, η^2^ = 0.007) and adjusted (F(6, 499376) = 355.27, *p* < 0.001, η^2^ = 0.004) analyses (see Figure 1A and Table 1). Participants with healthy diet scores had better healthy sleep scores in both models (see Table 2 for post hoc comparisons for unadjusted and adjusted analyses). No other statistically significant differences were observed.

#### 3.1.2. Associations between Food Groups and Sleep 

In order to examine the specific contributions of food groups and items to healthy sleep score, separate linear regression analyses were performed. In model 1 (unadjusted), the association between food group intake (vegetable, fruit, fish, unprocessed red meat, and processed meat) and healthy sleep score was analysed. In model 2, age, sex, and BMI were added as covariates in the linear model described in model 1. In model 3, mental health symptomatology was added to model 2. Model 4 (unadjusted) examined the associations between liquid intake (tea, coffee, and water) and healthy sleep score, which was then adjusted for age, sex, and BMI in model 5 and further adjusted for mental health symptomatology in model 6.

Results from statistical models are represented in Table 3. Model 1 revealed a statistically significant association between healthy sleep score and food group intake, F(5, 482813) = 799.41, *p* < 0.001, R^2^(adj) = 0.008, with better healthy sleep scores associated with increased vegetable, fruit and fish, but lower intake of unprocessed red meat and processed meat. In model 2, this association was significant again, F(8, 482818) = 2695.39, *p* < 0.001, R^2^(adj) = 0.043, with better healthy sleep scores associated with increased intake of vegetable, fruit, fish, and unprocessed red meat, but lower intake of processed meat. Model 3 revealed a significant association, F(9, 482818) = 5419.05, *p* < 0.001, R^2^(adj) = 0.092, with better healthy sleep scores associated with increased intake of vegetable, fruit, and fish, but lower intake of processed meat.

Model 4 (F(3, 417812) = 320.73, *p* < 0.001, R^2^(adj) = 0.002), Model 5 (F(6, 417812) = 2821.19, *p* < 0.001, R^2^(adj) = 0.039), and Model 6 (F(7, 417963) = 5890.55, *p* < 0.001, R^2^(adj) = 0.090) revealed significant associations between healthy sleep score and liquid intake, where increased healthy sleep scores were associated with increased water but lower intakes of tea and coffee.

#### 3.1.3. Fibre, Milk, and Sleep

In order to examine the associations between fibre intake and healthy sleep score, we conducted univariate ANOVA and ANCOVA (adjusted for age, sex, BMI) with partial fibre score as an independent variable and mental health symptomatology as a dependent variable. In both unadjusted, F(4, 501188) = 631.39, *p* < 0.001, η^2^= 0.005, and adjusted, F(4, 498,489) = 699.23, *p* < 0.001, η^2^ = 0.00, analyses, we observed a significant effect of fibre intake on healthy sleep score (see Figure 1B and Table 1). In both unadjusted and adjusted analyses, the low fibre intake group had reduced healthy sleep score compared to low/medium, medium, medium/high, and high fibre intake groups. Additionally, the low/medium fibre intake group had reduced healthy sleep score compared to medium, medium/high, and high fibre intake groups. Finally, only in the adjusted model, we observed that medium fibre intake group had lower healthy sleep scores compared to high fibre intake group. No other statistically significant differences were observed.

We also conducted univariate ANOVA and ANCOVA (adjusted for age, sex, BMI) with milk intake as an independent variable and healthy sleep score as a dependent variable to examine the effect of milk intake on sleep. These analyses revealed a significant main effect of milk intake on healthy sleep score both in the unadjusted, F(4, 484353) = 128.12, *p* < 0.001, η^2^ = 0.001, and adjusted model, F(4, 481773) = 120.84, *p* < 0.001, η^2^ = 0.001 (See Figure 1C and Table 1). In both unadjusted and adjusted analyses, we observed an inverted U relationship, showing that both low and high milk intake groups had lower healthy sleep scores compared to low/medium, medium, and medium/high milk intake groups (see Table 2 for post hoc analyses). No other statistically significant differences were observed.

Additionally, quadratic regression analyses were performed to quantify the nonlinear associations between healthy sleep score and (i) fibre and (ii) milk intakes and significant quadratic relationships were observed in both unadjusted (fibre: F(2, 498496) = 1123.39, *p* < 0.001, R^2^(adj) = 0.004; milk: F(2, 481780) = 115.99, *p* < 0.001, R^2^(adj) = 0.0005) and adjusted (for age, sex, BMI) (fibre: F(5, 498496) = 4442.30, *p* < 0.001, R^2^(adj) = 0.043; milk: F(5, 481780) = 3814.02, *p* < 0.001, R^2^(adj) = 0.038) analyses.

### 3.2. Diet and Mental Health

#### 3.2.1. Associations between Quality on Mental Health

**Figure 2:**
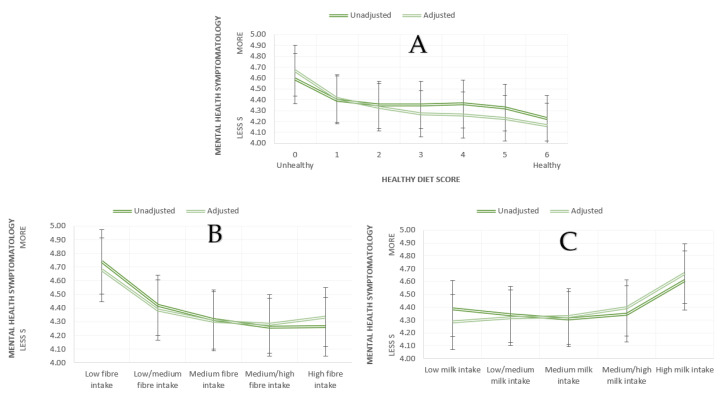
Mean mental health symptomatology according to (**A**) healthy diet scores (**B**) fibre intake, (**C**) milk intake (bars represent 95% CI).

After excluding diet score of seven due to low number of participants (n = 1) from the analyses, we conducted univariate ANOVA and ANCOVA (adjusted for age, sex, BMI) with healthy diet score as an independent variable and mental health symptomatology as a dependent variable. We observed a significant main effect of healthy diet score on mental health symptomatology in the unadjusted (F(6, 501228) = 49.10, *p* < 0.001, η^2^ = 0.001) and adjusted model (F(6, 498,533) = 124.17, *p* < 0.001, η^2^ = 0.001) (see Figure 2A and Table 4). Participants with healthy diet scores reported fewer mental health symptoms in both models (see Table 5 for post hoc comparisons for unadjusted and adjusted analyses). No other statistically significant differences were observed.

#### 3.2.2. Associations between Food Groups and Mental Health

To examine the specific contributions of food groups and items to the mental health symptomatology, separate linear regression analyses were performed. In model 1 (unadjusted), the association between food group intake (vegetable, fruit, fish, unprocessed red meat, and processed meat) and mental health symptomatology was analysed. In model 2, age, sex, and BMI were added as covariates in the linear model described in model 1. In model 3, healthy sleep score was added to model 2. Model 4 (unadjusted) examined the associations between liquid intake (tea, coffee, and water) and mental health symptomatology, which was then adjusted for age, sex, and BMI in model 5 and further adjusted for healthy sleep score in model 6. 

Results from statistical models are represented in Table 6. Model 1 revealed a statistically significant association between mental health symptomatology and food group intake, F(5, 482818) = 532.91, *p* < 0.001, R^2^(adj) = 0.005, with higher number of mental health symptoms associated with lower vegetable, fruit, fish, and unprocessed red meat intake, but higher processed meat intake. This association was again significant in model 2, F(8, 482818) = 2084.64, *p* < 0.001, R^2^(adj) = 0.033, and in model 3, F(9, 482818) = 4846.88, *p* < 0.001, R^2^(adj) = 0.083. Lower intake of vegetables, fruits, fish, and unprocessed red meat and higher intake of processed meat were associated with a higher number of mental health symptoms.

Although model 4 revealed that a higher number of mental health symptoms were associated with higher intake of tea, coffee, and water, F(3, 417812) = 349.88, *p* < 0.001, R^2^(adj) = 0.002, when adjusted for age, sex, and BMI, model 5 showed that a higher number of mental health symptoms was associated with lower water intake but higher tea and coffee intake, F(6, 417812) = 2242.75, *p* < 0.001, R^2^(adj) = 0.031. In model 6, after further adjusting for mental health symptomatology, the associations observed in model 5 were replicated, F(7, 417812) = 5367.03, *p* < 0.001, R^2^(adj) = 0.082.

#### 3.2.3. Fibre, Milk, and Mental Health

In order to examine the associations between fibre intake and mental health, we conducted univariate ANOVA and ANCOVA (adjusted for age, sex, BMI) with partial fibre score as an independent variable and mental health symptomatology as a dependent variable. We observed a significant main effect of partial fibre score on mental health symptomatology, F(4, 500978) = 388.02, *p* < 0.001, η^2^= 0.003 (see Figure 2B and Table 4). Both the high and medium/high fibre intake groups had lower numbers of mental health symptoms compared to medium, medium/low, and low fibre intake groups. The medium fibre intake group had lower numbers of mental health symptoms compared to both the low/medium and low fibre intake groups and the low/medium fibre intake group reported a lower number of mental health symptoms compared to the low fibre intake group (see Table 5 for post hoc comparisons). The adjusted model revealed a significant effect of partial fibre score on mental health symptomatology, F(4, 498296) = 257.36, *p* < 0.001, η^2^ = 0.002 (see Figure 2B). The high, medium/high, and medium fibre intake groups had a lower number of mental health symptomatology compared to the low/medium and low fibre intake groups. The low/medium fibre intake group reported to have less mental health symptoms compared to the low fibre intake group. Interestingly, the high fibre intake group also had an increased number of mental health symptoms compared to the medium/high fibre intake group (see Table 5 for post hoc comparisons). No other statistically significant differences were observed.

We also conducted univariate ANOVA and ANCOVA (adjusted for age, sex, BMI) with milk intake as an independent variable and mental health symptomatology as a dependent variable to examine the effect of milk intake on mental health. We observed a significant main effect of milk intake on mental health symptomatology, F(4, 484,144) = 133.60, *p* < 0.001, η^2^ = 0.0031 (See Figure 2C and Table 4). The high milk intake group reported higher numbers of mental health symptoms compared to the medium/high, medium, medium/low, and low milk intake groups. Additionally, both the medium and low/medium milk intake groups reported higher numbers of mental health symptoms compared to the low milk intake group (see Table 5 for post hoc comparisons). Similarly, in the adjusted model, the effect of milk intake on mental health symptomatology was significant F(4, 481,577) = 223.73, *p* < 0.001, η^2^ = 0.002 (see Figure 2C and Table 4). Post hoc analyses revealed that medium, medium/high, and high milk intake groups reported more mental health symptoms compared to low milk intake group. High and medium/high had increased number of mental health symptoms compared to both the low/medium and medium milk intake groups. Finally, the high milk intake group reported more mental health symptoms compared to the medium/high milk intake group (see Table 5 for post hoc comparisons). No other statistically significant differences were observed.

In addition, quadratic regression analyses were performed to quantify the nonlinear associations between mental health symptomatology and (i) fibre and (ii) milk intakes and significant quadratic relationships were observed in both unadjusted (fibre: F(2,498303) = 690.22, *p* < 0.001, R^2^(adj) = 0.003; milk: F(2, 481582) = 214.19, *p* < 0.001, R^2^(adj) = 0.001) and adjusted (for age, sex, BMI) (fibre: F(5, 498303) = 3103.60, *p* < 0.001, R^2^(adj) = 0.030; milk: F(5, 481584) = 3064.68, *p* < 0.001, R^2^(adj) = 0.031) analyses.

## 4. Discussion

In this large contemporary prospective study of half a million men and women from the UK, we examined the associations of healthy diet and food groups/items with healthy sleep/sleep quality and mental health symptomatology. We observed positive associations between healthy diet and (i) sleep and (ii) mental health, especially benefits of high intakes of vegetable, fruit, fish, water, and fibre. On the other hand, we showed inverse associations between processed meat and milk intake and sleep and mental health. We also highlighted the role of (i) sleep in mental health and diet relationship and (ii) mental health in sleep and diet relationship.

### 4.1. Healthy Diet and Food Groups

In accordance with previous research showing that adults in the UK consume insufficient amounts of vegetables, fruits, fish, and fibre, but excess amounts of saturated fats, sugar, unprocessed and processed red meat, and sugar-sweetened beverages [56,57], we found that adherence to a healthy/good-quality diet is very low, with only 7.9% of the participants adhering to a healthy/high-quality diet. Both in unadjusted and age, sex, BMI-adjusted analyses, we found that people who scored higher on the healthy diet measure also scored higher on healthy sleep/sleep quality and lower on mental health symptomology, although the association between healthy diet score and mental health symptomatology was magnified when adjusted. These findings (i) extend the very limited evidence showing positive associations between Mediterranean-style diet and sleep quality [34], (ii) add to the current evidence suggesting that diet quality and adherence to a Mediterranean-style diet may play a role in depression and anxiety risk and severity [25,38,39,40,41], and (iii) suggest that dietary interventions addressing mental health as a whole, rather than focusing on the symptomatology or specific diagnoses that comprises it, may be a useful means of improving population’s general mental health and wellbeing. 

When focusing on food groups, although a recent study showed an association between sleeping the recommended duration (7–9 h/day) and vegetable and fruit consumption [58], to the best of our knowledge, the current study is the first to analyse the associations between various food groups and healthy sleep/sleep quality. In an unadjusted model, we showed that higher healthy sleep scores were associated with increased vegetable, fruit, and fish, but decreased unprocessed red meat and processed meat intake. However, when controlled for age, sex, and BMI, both better healthy sleep scores and fewer reported mental health symptoms were associated with increased vegetable, fruit, fish, and unprocessed red meat, but less consumption of processed meat. The same pattern was also observed for unadjusted and further healthy sleep-adjusted analyses for food groups and mental health symptomatology. Additionally, after further controlling for mental health symptomatology, we showed that unprocessed red meat was no longer associated with better sleep; however, positive associations between sleep and vegetable, fruit, fish intakes and the negative association between sleep and processed meat intake were replicated. As healthy/high-quality/Mediterranean-style diets are characterised by higher intakes of vegetable, fruit, and unprocessed lean protein, these results, again, could be explained by the benefits of adhering to a healthy/good quality/Mediterranean-style diet [25,34,38,39,40,41]. Additionally, the direction of our results for processed meat consumption supports previous research that shows negative associations between habitual meat consumption (processed and unprocessed red meat) and sleep duration and quality [59]. This might also be explained by reduced ratios of tryptophan and tyrosine (and their capacity to synthesize melatonin, serotonin, and dopamine) leading to a reduction in the synthesis of brain sleep inductors and, hence, to a deterioration of sleep parameters [60]. 

It is well known that both healthy/high-quality/Mediterranean-style diets and food groups that are abundant in such diets, specifically vegetables and fruits, apart from vitamins and minerals, contain substantial amounts of fibre and polyphenols, which are known to have anti-inflammatory, neuroprotective, and prebiotic properties [61,62] and also are associated with reduced systemic inflammation markers [63,64,65]. On the other hand, unhealthy dietary patterns and food groups (such as processed meats) are rich in saturated- and trans-fatty acids, which are known to be associated with alterations in the gut microbiota that could lead to an increased circulation of inflammatory markers [32,66]. Additionally, proinflammatory diets and higher levels of inflammatory biomarkers have been shown to be associated with shorter sleep duration [67] and increased risk of depression, anxiety, and lower likelihood of wellbeing [68]. Given the importance of gut microbiota diversity in utilising nutrients [69] and driving inflammation [70], improving gut microbiota diversity via anti-inflammatory dietary interventions in order to reduce inflammation may alter endocrine and neurotransmitter concentrations and lead to positive sleep and mental health outcomes.

Interestingly, we also observed that in both unadjusted and adjusted models for healthy sleep and adjusted models for mental health symptomatology, increased water but decreased tea and coffee intake were associated with better sleep and mental health. When these models further adjusted for mental health (for healthy sleep) and sleep (for mental health symptomatology), these associations became stronger. These results might reflect sleep- and mental health-related benefits of hydration. Convergent evidence comes from the studies investigating the effect of hydration on sleep duration and mood, in which short sleep duration was found to be associated with higher odds of inadequate hydration [71] and dehydration was found to acutely degrade mood, mainly in women [72]. Alternatively, although the stress-related health benefits of acute tea consumption have already been established due to the consumption of actives in the tea, such as l-theanine and epigallocatechin gallate [73,74], the negative associations we observed might also be explained by the harmful effects of habitual caffeine consumption on sleep quality [75,76] and mental health [77].

### 4.2. Fibre and Milk

Examining fibre in isolation showed that in both unadjusted and adjusted analyses, fibre intake was positively associated with healthy sleep scores and negatively associated with mental health symptomatology. We found that lowest fibre intake group had the lowest healthy sleep/sleep quality score, but the highest number of mental health symptomatology reported, followed by low/medium, medium, and high intake groups, except slightly increased total number of mental health symptoms in high fibre group after controlling for age, sex, and BMI. Although previous researchers have started linking fibre intake with sleep by showing associations between high fibre intake (i) and slower wave sleep and less time spent in stage 1 sleep [78] and (ii) sleep duration in adolescents [79], to the best of our knowledge, we are the first to show an association between fibre intake and sleep health/quality in an adult population. On the other hand, our mental health symptomatology results extend and contribute to previous findings showing (i) a potential role of dietary fibre in reducing the severity of depressive symptoms [80] and (ii) a positive association between dietary fibre intake and mental health quality of life scores [81]. Given the role of dietary fibre consumption in lowering inflammation by modifying both the pH and the permeability of the gut [80] and the role of gut–brain–axis in circadian rhythms [24] and mental health [23], inflammation may be a potential mediator between dietary fibre and sleep and mental health. Thus, increased dietary fibre intake may be beneficial for reducing or preventing inflammation (producing by-products that are beneficial for proper brain functioning), leading to an improvement in sleep and mental health outcomes. Although supporting evidence comes from studies showing heightened levels of inflammatory markers after sleep loss [82] and in depression and anxiety [68], this area of research is still in its infancy. Therefore, randomised-controlled trials are warranted to examine the (i) effect of source of fibre, (ii) effect of fibre amount, and (ii) role of inflammation on sleep and mental health outcomes.

Our milk intake findings showed an inverted-U-like relationship in both unadjusted and adjusted models, where both participants in the lowest and highest milk intake groups had the lowest healthy sleep/sleep quality scores. We also demonstrated that the highest milk intake group reported increased numbers of mental health symptoms. As milk contains 18 of 22 essential nutrients, including vitamin D, calcium, and various group B vitamins of especial importance for sleep (see reviews [20,35,36]), it is clear why lower milk intake is associated with poor sleep health/quality. However, milk is also the main dietary source of D-galactose [83,84], and chronic exposure to milk, with a dose corresponding to 1–2 glasses of milk in humans, is known to cause inflammation in animals [83]. Another factor that might contribute to inflammation is the dietary saturated fatty acid content of milk and dairy products, which, again, is known to modulate gut microbiota composition and low-grade systemic inflammation [85]. Given the role of inflammation in modulating circadian rhythms [24,86] and mental health [87], together these findings raise the possibility that higher milk intake, hence D-galactose and saturated fat intake, may cause inflammation and exacerbate sleep health and mental health symptoms. Furthermore, in the current study, derivation of the estimate of milk intake [54] were based on the questions on type of milk, bowls of breakfast cereal, cups of tea, and cups of coffee; therefore, these findings might also be partially attributable to the harmful effects of habitual caffeine consumption on sleep [75] and mental health [77] outcomes. 

### 4.3. Strengths and Limitations

Strengths of this study include the large number of participants and the ability to adjust for known and potential confounders. However, it is important to note that we were not able to adjust for total energy intake because, due to the nature of UKB food frequency questionnaire, estimated total energy intake was not available. Importantly, we adjusted for BMI, which has been shown to better approximate objectively measured total energy expenditure (and, therefore, true energy intake) than estimated energy intake from a food-frequency questionnaire [88]. 

On the other hand, we also wish to identify several important caveats. First of all, although UKB represent a large and unique resource, the sample was restricted to middle-aged and mostly European descent adults. Additionally, the recruitment method and low response rate (5.5%) was subject to selection bias [89]. All these factors may affect the generalisability of the results to other broader UK and other populations. Secondly, as this was a cross-sectional study, the relationships between diet, sleep, and mental health are not necessarily causal. Thirdly, although the diet, sleep, and mental health-related measures we utilised were single item subjective self-report questions, the misclassification of exposures is unavoidable. Such misclassifications would be expected to attenuate findings toward the null. Fourthly, for simplicity, we used healthy diet score, healthy sleep score, and total number of mental health symptoms reported by dichotomising various factors, which may result in loss of information and study power. Fifthly, we also estimated fibre and milk intake based on participant’s answers to other related questions, and although moderate to substantial agreement between the responses to the dietary touchscreen questions at baseline and the repeat visit has been observed for fibre intake score [53], milk intake score did not contain all sources of milk [54] and, hence, is prone to a measurement error in milk intake estimation. Sixthly, sample sizes were different across the analyses due to missing data for those specific analyses. Seventhly, although significances were observed, the effect sizes we reported were small to moderate. Finally, although we calculated the total number mental health symptoms reported as a marker of mental health symptomatology, this has not been validated; therefore, further research is warranted to examine the agreement between the total number of mental health symptoms reported in the UKB and the actual mental health status.

## 5. Conclusions

In conclusion, we found that although adherence to a healthy dietary pattern is low, a healthy dietary pattern, and food groups (vegetables, fruits, fish, water) and nutrients (fibre) that are consumed as a part of a healthy dietary pattern were associated with better sleep quality and mental health. Aside from the aforementioned suggestions, future research is needed to identify underlying mechanisms behind the impact of diet on sleep and mental health and the role of inflammation and gut health, by utilising RCTs, preferably by using objective measures of sleep and mental health, in a diverse sample across time, in (i) clinical samples to determine the most cost-effective and sustainable methods for providing dietary interventions as a primary or complementary method of treatment and (ii) subclinical samples to develop and evaluate population sleep and mental wellbeing strategies. Given the association between sleep health and mental health symptomatology, treatments and improvement strategies should not be considered in isolation.

## Figures and Tables

**Table 1:** Unadjusted and covariate adjusted descriptive statistics for healthy sleep scores across healthy diet score, partial fibre, and milk intake groups.

		*N*	Mean ± SD	Mean _adj._ (SE)
**Healthy Diet Score**	0 (unhealthy)	65723	2.59 ± 1.15	2.64 (0.004)
	1	165603	2.77 ± 1.10	2.78 (0.003)
	2	149435	2.84 ± 1.10	2.84 (0.003)
	3	81980	2.88 ± 1.09	2.86 (0.004)
	4	31207	2.92 ± 1.10	2.88 (0.006)
	5	7688	2.96 ± 1.11	2.90 (0.012)
	6 (healthy)	856	2.94 ± 1.11	2.88 (0.037)
**Partial Fibre Groups**	Low	100303	2.65 ± 1.12	2.65 (0.003)
	Low/medium	100199	2.81 ± 1.10	2.80 (0.003)
	Medium	100024	2.85 ± 1.09	2.85 (0.003)
	Medium/high	100386	2.86 ± 1.09	2.86 (0.003)
	High	100281	2.85 ± 1.11	2.87 (0.003)
**Milk Intake Groups**	Low	96543	2.77 ± 1.11	2.76 (0.004)
	Low/medium	100710	2.83 ± 1.10	2.83 (0.003)
	Medium	94754	2.83 ± 1.09	2.84 (0.004)
	Medium/high	95455	2.84 ± 1.09	2.84 (0.004)
	High	96896	2.76 ± 1.12	2.77 (0.003)

*adj*: adjusted for ages, sex, BMI.

**Table 2:** Post hoc comparisons for unadjusted and covariate adjusted analyses using Bonferroni. Mean differences shown. * shows mean difference is significant at the 0.05 level.

	Unadjusted							Adjusted						
	Healthy Diet Score							Healthy Diet Score						
	0	1	2	3	4	5	6	0	1	2	3	4	5	6
0	1	−0.18 *	−0.24 *	−0.29 *	−0.33 *	−0.37 *	−0.35 *	1	−0.15 *	−0.20 *	−0.23 *	−0.24 *	−0.27 *	−0.24 *
1		1	−0.07 *	−0.11 *	−0.15 *	−0.19 *	−0.17 *		1	−0.06 *	−0.08 *	−0.10 *	−0.12 *	−0.09
2			1	−0.05 *	−0.09 *	−0.12 *	−0.10			1	−0.03 *	−0.04 *	−0.07 *	−0.04
3				1	−0.04 *	−0.08 *	−0.06				1	−0.02	−0.04 *	−0.01
4					1	−0.04	−0.02					1	−0.02	0.00
5						1	0.02						1	0.02
6							1							1
	**Partial Fibre Groups**							**Partial Fibre Groups**						
	1		2	3	4	5		1		2	3	4	5	
1. Low	1		−0.15 *	−0.20 *	−0.20 *	−0.20 *		1		−0.15 *	−0.20 *	−0.21 *	−0.22 *	
2. Low/medium			1	−0.05 *	−0.05 *	−0.05 *				1	−0.05 *	−0.06 *	−0.07 *	
3. Medium				1	−0.00	−0.00					1	−0.01	−0.02 *	
4. Medium/high					1	0.00						1	−0.00	
5. High						1							1	
	**Milk Intake Groups**							**Milk Intake Groups**						
	1		2	3	4	5		1		2	3	4	5	
1. Low	1		−0.07 *	−0.07 *	−0.07 *	0.00		1		−0.07 *	−0.07 *	−0.08 *	−0.00	
2. Low/medium			1	0.00	−0.00	0.08 *				1	−0.00	−0.00	0.06 *	
3. Medium				1	−0.00	0.08 *					1	−0.00	0.07 *	
4. Medium/high					1	0.08 *						1	0.07 *	
5. High						1							1	

**Table 3:** Regression analysis summary for healthy sleep score.

Model		B	SE	β	95% CI	*t*	*p*	R^2^	R^2^(adj)	Cohen’s f^2^
1								0.008	0.008	0.008
	(Constant)	2.874	0.006		2.862, 2.886	480.117	0.000			
	Vegetable intake	0.001	0.000	0.004	0.000, 0.002	2.566	0.010			
	Fruit intake	0.011	0.001	0.025	0.009, 0.012	16.811	0.000			
	Fish intake	0.021	0.001	0.028	0.019, 0.023	18.864	0.000			
	Unprocessed red meat intake	−0.009	0.001	−0.014	−0.011, −0.007	−9.179	0.000			
	Processed meat intake	−0.073	0.002	−0.070	−0.076, −0.070	−44.996	0.000			
2								0.043	0.043	0.044
	(Constant)	4.429	0.014		4.401, 4.457	310.269	0.000			
	Age	−0.011	0.000	−0.078	−0.011, −0.010	−54.412	0.000			
	Sex (F = 0/M = 1)	−0.053	0.003	−0.024	−0.059, −0.046	−16.212	0.000			
	BMI	−0.038	0.000	−0.164	−0.038, −0.037	−115.352	0.000			
	Vegetable intake	0.002	0.000	0.007	0.001, 0.003	4.905	0.000			
	Fruit intake	0.011	0.001	0.027	0.010, 0.012	17.769	0.000			
	Fish intake	0.026	0.001	0.034	0.024, 0.028	23.754	0.000			
	Unprocessed red meat intake	0.004	0.001	0.006	0.002, 0.006	3.881	0.000			
	Processed meat intake	−0.053	0.002	−0.051	−0.056, −0.050	−32.155	0.000			
3								0.092	0.092	0.100
	(Constant)	4.962	0.014		4.934, 4.990	347.16	0.000			
	Age	−0.014	0.000	−0.101	−0.014, −0.013	−71.628	0.000	0.015	0.015	0.007
	Sex (F = 0/M = 1)	−0.121	0.003	−0.055	−0.127, −0.115	−37.741	0.000	0.016	0.016	0.0008
	BMI	−0.036	0.000	−0.158	−0.037, −0.036	−114.032	0.000	0.026	0.026	0.027
	Mental health Symptomatology	−0.077	0.000	−0.225	−0.078, −0.076	−161.385	0.000	0.049	0.049	0.051
	Vegetable intake	0.001	0.000	0.004	0.000, 0.002	2.710	0.007	0.001	0.001	0.0005
	Fruit intake	0.010	0.001	0.023	0.008, 0.011	15.626	0.000	0.002	0.002	0.001
	Fish intake	0.021	0.001	0.027	0.018, 0.023	19.068	0.000	0.003	0.003	0.0005
	Unprocessed red meat intake	−0.002	0.001	−0.003	−0.004, 0.000	−1.880	0.060	0.004	0.004	0.001
	Processed meat intake	−0.041	0.002	−0.039	−0.044, -0.037	−25.366	0.000	0.008	0.008	0.004
4								0.002	0.002	0.002
	(Constant)	2.890	0.004		2.881, 2.898	650.149	0.000			
	Tea intake	−0.012	0.001	−0.032	−0.014, −0.011	−20.018	0.000			
	Coffee intake	−0.021	0.001	−0.040	−0.023, −0.020	−24.944	0.000			
	Water intake	0.005	0.001	0.011	0.004, 0.007	6.951	0.000			
5								0.039	0.039	0.040
	(Constant)	4.475	0.016		4.445, 4.506	288.374	0.000			
	Age	−0.008	0.000	−0.062	−0.009, −0.008	−40.425	0.000			
	Sex (F = 0/M = 1)	−0.088	0.003	−0.039	−0.094, −0.081	−25.730	0.000			
	BMI	−0.039	0.000	−0.171	−0.040, −0.039	−112.166	0.000			
	Tea intake	−0.011	0.001	−0.029	−0.012, −0.010	−18.425	0.000			
	Coffee intake	−0.015	0.001	−0.029	−0.017, −0.014	−18.148	0.000			
	Water intake	0.003	0.001	0.005	0.001, 0.004	3.363	0.001			
6								0.090	0.090	0.098
	(Constant)	4.989	0.015		4.958, 5.019	322.467	0.000			
	Age	−0.012	0.000	−0.088	−0.012, −0.012	−58.970	0.000	0.005	0.005	0.004
	Sex (F = 0/M = 1)	−0.151	0.003	−0.068	−0.158, −0.145	−45.243	0.000	0.003	0.003	0.002
	BMI	−0.038	0.000	−0.164	−0.038, −0.037	−110.441	0.000	0.029	0.029	0.029
	MH Sym.	−0.079	0.001	−0.229	−0.080, −0.078	−152.841	0.000	0.051	0.051	0.053
	Tea intake	−0.006	0.001	−0.016	−0.007, −0.005	−10.420	0.000	0.001	0.001	0.0005
	Coffee intake	−0.012	0.001	−0.023	−0.014, −0.011	−15.128	0.000	0.002	0.002	0.001
	Water intake	0.002	0.001	0.004	0.000, 0.003	2.628	0.009	0.000	0.000	0.0001

MH: mental health; Sym: symptomatology.

**Table 4:** Unadjusted and covariate adjusted descriptive statistics for mental health symptomatology across healthy diet score, partial fibre, and milk intake groups.

		*N*	Mean ± SD	Mean _adj._ (SE)
**Healthy Diet Score**	0 (unhealthy)	64,650	4.59 ± 3.30	4.68 (0.01)
	1	165,528	4.40 ± 3.22	4.44 (0.008)
	2	149,364	4.35 ± 3.19	4.34 (0.008)
	3	81,955	4.35 ± 3.19	4.28 (0.01)
	4	31,198	4.36 ± 3.22	4.26 (0.01)
	5	7685	4.32 ± 3.25	4.23 (0.03)
	6 (healthy)	855	4.23 ± 3.12	4.16 (0.10)
**Partial Fibre Groups**	Low	100,207	4.74 ± 3.31	4.68 (0.01)
	Low/medium	100,168	4.42 ± 3.21	4.39 (0.01)
	Medium	99,997	4.32 ± 3.17	4.31 (0.01)
	Medium/high	100,366	4.26 ± 3.17	4.28 (0.01)
	High	100,245	4.26 ± 3.21	4.34 (0.01)
**Milk Intake Groups**	Low	96,441	4.39 ± 3.25	4.29 (0.01)
	Low/medium	100,674	4.34 ± 3.18	4.32 (0.01)
	Medium	94,725	4.31 ± 3.18	4.33 (0.01)
	Medium/high	95430	4.35 ± 3.18	4.40 (0.01)
	High	96,875	4.61 ± 3.29	4.66 (0.01)

*adj*: adjusted for ages, sex, BMI.

**Table 5:** Post hoc comparisons for unadjusted and covariate adjusted analyses using Bonferroni. Mean differences shown. * shows mean difference is significant at the 0.05 level.

	Unadjusted							Adjusted						
	Healthy Diet Score							Healthy Diet Score						
	0	1	2	3	4	5	6	0	1	2	3	4	5	6
0	1	0.20 *	0.24 *	0.24 *	0.23 *	0.27 *	0.36 *	1	0.23 *	0.34 *	0.40 *	0.42 *	0.44 *	0.52 *
1		1	−0.05 *	0.05 *	0.04	0.07	0.17		1	0.11 *	0.16 *	0.18 *	0.21 *	0.28
2			1	0.00	−0.01	0.03	0.12			1	0.06 *	0.08 *	0.10	0.18
3				1	−0.01	0.02	0.12				1	0.02	0.04	0.12
4					1	0.03	0.13					1	0.03	0.10
5						1	0.10						1	0.07
6							1							1
	**Partial Fibre Groups**							**Partial Fibre Groups**						
	1	2	3		4	5		1	2	3		4	5	
1. Low	1	0.32 *	0.42 *		0.48 *	0.48 *		1	0.30 *	0.38 *		0.40 *	0.34 *	
2. Low/medium		1	0.10 *		0.16 *	0.16 *			1	0.08 *		0.10 *	0.05 *	
3. Medium			1		0.06 *	0.05 *				1		0.02	−0.03	
4. Medium/high					1	−0.00						1	−0.05 *	
5. High						1							1	
	**Milk Intake Groups**							**Milk Intake Groups**						
	1	2	3		4	5		1	2	3		4	5	
1. Low	1	0.05 *	0.08 *		0.04	−0.22 *		1	−0.04	−0.04 *		−0.11 *	−0.38 *	
2. Low/medium		1	0.04		−0.00	−0.26 *			1	-0.00		−0.08 *	−0.34 *	
3. Medium			1		−0.04	−0.30 *				1		−0.07 *	−0.34 *	
4. Medium/high					1	−0.26 *						1	−0.27 *	
5. High						1							1	

**Table 6:** Regression analysis summary for mental health symptomatology.

Model		B	SE	β	95% CI	*t*	*p*	R^2^	R^2^(adj)	Cohen’s f^2^
1								0.005	0.005	0.005
	(Constant)	5.057	0.017		5.023, 5.091	289.220	0.000			
	Vegetable intake	−0.012	0.001	−0.012	−0.014, −0.009	−8.081	0.000			
	Fruit intake	−0.023	0.002	−0.018	−0.026, −0.019	−12.128	0.000			
	Fish intake	−0.099	0.003	−0.044	−0.106, −0.093	−30.318	0.000			
	Unprocessed red meat intake	−0.090	0.003	−0.050	−0.096, −0.085	−32.254	0.000			
	Processed meat intake	0.068	0.005	0.023	0.059, 0.078	14.496	0.000			
2								0.033	0.033	0.034
	(Constant)	6.894	0.042		6.812, 6.976	164.757	0.000			
	Age	−0.040	0.001	−0.100	−0.041, −0.039	−69.194	0.000			
	Sex (F = 0/M = 1)	−0.882	0.010	−0.137	−0.901, −0.863	−92.381	0.000			
	BMI	0.018	0.001	0.027	0.016, 0.020	18.652	0.000			
	Vegetable intake	−0.014	0.001	−0.015	−0.017, −0.011	−9.999	0.000			
	Fruit intake	−0.021	0.002	−0.017	−0.025, −0.018	−11.512	0.000			
	Fish intake	−0.074	0.003	−0.033	−0.081, −0.068	−22.832	0.000			
	Unprocessed red meat intake	−0.070	0.003	−0.039	−0.076, −0.065	−25.315	0.000			
	Processed meat intake	0.157	0.005	0.052	0.148, 0.167	32.674	0.000			
3								0.083	0.083	0.090
	(Constant)	9.83	0.045		9.743, 9.918	220.24	0.000			
	Age	−0.047	0.001	−0.118	−0.048, −0.046	−83.429	0.000	0.010	0.010	0.010
	Sex (F = 0/M = 1)	−0.917	0.009	−0.142	−0.935, −0.899	−98.573	0.000	0.017	0.017	0.017
	BMI	−0.007	0.001	−0.011	−0.009, −0.005	−7.536	0.000	0.001	0.001	0.0006
	Healthy Sleep Score	−0.663	0.004	−0.227	−0.671, −0.655	−161.385	0.000	0.049	0.049	0.0520
	Vegetable intake	−0.013	0.001	−0.013	−0.015, −0.010	-9.126	0.000	0.001	0.001	0.0006
	Fruit intake	−0.014	0.002	−0.011	−0.017, −0.010	−7.675	0.000	0.000	0.000	0.0004
	Fish intake	−0.057	0.003	−0.025	−0.063, −0.051	−17.913	0.000	0.002	0.002	0.0022
	Unprocessed red meat intake	−0.068	0.003	−0.037	−0.073, −0.063	−25.084	0.000	0.002	0.002	0.0017
	Processed meat intake	0.122	0.005	0.040	0.113, 0.131	26.053	0.000	0.000	0.000	0.0004
4								0.003	0.002	0.002
	(Constant)	4.067	0.013		4.042, 4.093	313.881	0.000			
	Tea intake	0.056	0.002	0.050	0.053, 0.060	31.025	0.000			
	Coffee intake	0.027	0.002	0.017	0.022, 0.032	10.826	0.000			
	Water intake	0.031	0.002	0.021	0.026, 0.035	13.665	0.000			
5								0.031	0.031	0.032
	(Constant)	6.514	0.045		6.425, 6.603	143.367	0.000			
	Age	−0.046	0.001	−0.115	−0.047, −0.045	−74.978	0.000			
	Sex (F = 0/M = 1)	−0.811	0.010	−0.125	−0.830, −0.791	−81.347	0.000			
	BMI	0.021	0.001	0.031	0.019, 0.023	20.060	0.000			
	Tea intake	0.065	0.002	0.058	0.061, 0.068	36.147	0.000			
	Coffee intake	0.037	0.002	0.024	0.032, 0.042	15.189	0.000			
	Water intake	-0.009	0.002	−0.006	−0.013, −0.004	−3.821	0.000			
6								0.083	0.082	0.089
	(Constant)	9.529	0.048		9.434, 9.624	196.818	0.000			
	Age	−0.051	0.001	−0.130	−0.053, −0.050	−86.456	0.000	0.013	0.013	0.013
	Sex (F = 0/M = 1)	−0.870	0.010	−0.134	−0.889, −0.851	−89.587	0.000	0.015	0.015	0.015
	BMI	−0.006	0.001	−0.009	−0.008, −0.004	−5.818	0.000	0.001	0.001	0.0009
	Healthy sleep score	−0.674	0.004	−0.231	−0.682, −0.665	−152.841	0.000	0.051	0.051	0.054
	Tea intake	0.057	0.002	0.051	0.054, 0.061	32.762	0.000	0.002	0.002	0.001
	Coffee intake	0.027	0.002	0.018	0.022, 0.032	11.295	0.000	0.000	0.000	0.0001
	Water intake	−0.007	0.002	−0.005	−0.011, −0.003	−3.114	0.002	0.000	0.000	0.0004

## Data Availability

The data that support the findings of this study are available from UK Biobank (http://www.ukbiobank.ac.uk/about-biobank-uk/ accessed on 18 June 2021). Restrictions apply to the availability of these data, which were used under license for the current study (Project ID: 61818). Data are available for bona fide researchers upon application to the UK Biobank.

## Supplementary Materials

The following are available online at https://www.mdpi.com/article/10.3390/nu13082573/s1. Table S1: Baseline characteristics according to healthy diet score; Table S2: Baseline characteristics according to partial fibre score; Table S3: Baseline characteristics according to milk intake estimations; Table S4: Baseline characteristics according to healthy sleep scores; Table S5: Baseline characteristics according to mental health symptomatology reported, Table S6: Correlations between mental health questions and total number of mental health symptomatology reported; Table S7: Cronbach’s Alpha Scores if total number of mental health symptomatology items deleted, Text S1: Supplementary Methods.
