# Supplementary material for: Diet, Sleep, and Mental Health: Insights from the UK Biobank Study
Source: Nutrients. 2021 Jul 27;13(8):2573. doi: 10.3390/nu13082573 (PMC8398967; doi:10.3390/nu13082573)
Supplement: Supplementary file 1 [file nutrients-13-02573-s001.zip › supp data.pdf]

Table S1. Baseline characteristics according to healthy diet score

	Healthy Diet Score							
	0	1	2	3	4	5	6	7
Number of participants	65723	165603	149435	81980	31207	7688	856	2
Sex								
Female (%)	36.2	47.4	59.1	66.8	70.0	71.6	72.9	100
Male (%)	63.8	52.6	40.9	33.2	30.0	28.4	27.1	0
Age (M ± SD)	55.1±8.3	56.2±8.1	57.0± 7.9	57.0±7.9	56.8±7.9	57.1±7.8	57.5± 7.4	48.5± 3.5
BMI (kg/m2)	28.0±4.9	27.6±4.7	27.3±4.7	26.9±4.7	26.5±4.6	26.0±4.5	25.8±4.4	24.6±2.9
Townsend Index (M ± SD)	-.8±3.3	-1.4±3.0	-1.4±3.0	-1.3±3.0	-1.1±3.1	-1.0±3.1	-.8± 3.3	-1.8± 2.7
Employment status (%)								
In paid employment or self-employed	62.1	58.8	56.1	55.9	56.9	56.2	52.4	100
Retired	27.0	32.7	35.9	36.0	34.8	34.5	38.4	0
Looking after home and/or family	2.4	2.7	2.8	3.0	3.0	3.0	3.0	0
Unable to work because of sickness or	5.3	3.4	3.0	2.9	2.9	3.5	3.7	0
disability	2.6	1.7	1.4	1.3	1.4	1.7	1.5	0
Unemployed	0.3	0.4	0.5	0.6	0.7	0.8	1.0	0
Doing unpaid or voluntary work	0.2	0.2	0.2	0.3	0.4	0.4	0.1	0
Full-time or part-time student								
Qualifications (%)								
College or University Degree	31.7	38.6	40.0	42.7	46.9	49.3	52.7	50.0
A levels/AS levels or equivalent	13.5	13.9	13.6	13.3	13.0	13.3	12.7	0
O levels/GCSEs or equivalent	29.1	26.3	25.7	24.5	22.5	20.9	20.2	50.0
CSEs or equivalent	9.6	6.9	6.2	5.6	4.9	4.1	3.4	0
NVQ or HND or HNC or equivalent	10.3	8.4	7.7	7.1	6.4	6.0	5.4	0
Other professional qualifications	5.7	5.9	6.8	6.8	6.3	6.4	5.6	0
Total household income (%)								
Less than 18,000	25.3	21.7	22.5	23.2	24.2	25.6	26.6	0
18,000-30,999	25.3	25.0	25.7	25.8	25.8	25.2	27.3	0
31,000-51,999	26.2	26.4	25.9	25.7	25.6	24.6	21.2	0
52,000-100,000	18.5	21.3	20.4	20.0	19.3	19.4	19.5	100
Greater than 100,000	4.6	5.6	5.6	5.3	5.1	5.2	5.4	0
Shift work (%)								
Never/rarely	79.2	82.9	83.5	83.6	83.3	82.4	84.5	100
Sometimes	8.7	7.3	7.1	7.1	7.6	7.9	7.8	0
Usually	2.6	2.1	2.0	2.1	2.1	2.3	1.4	0
Always	9.6	7.7	7.4	7.1	7.0	7.4	6.4	0
Night shift work (%)								

Never/rarely	45.7	48.8	49.6	50.8	54.0	53.4	55.7	0
Sometimes	29.9	28.4	28.9	28.3	26.2	27.6	22.9	0
Usually	8.0	8.5	7.7	7.2	7.3	6.1	8.6	0
Always	16.4	14.4	13.7	13.7	12.6	12.9	12.9	0

Table S2. Baseline characteristics according to partial fibre score

	**Partial Fibre Score (Quintiles)**				
	**Low**	**Low/medium**	**Medium**	**Medium/high**	**High**
Number of participants	100303	100199	100024	100386	100281
Mean ± SD	8.00±2.24	12.26±0.82	14.90±0.73	17.71±0.93	24.12±6.15
Sex					
Female (%)	50.2	55.2	57.5	57.1	52.1
Male (%)	49.8	45.8	42.5	42.9	47.9
Age (M ± SD)	54.5±8.1	55.9±8.1	56.8±8.0	57.3±7.8	57.7±7.8
BMI (kg/m2)	27.6±4.9	27.4±4.7	27.3±4.7	27.2±4.7	27.3±4.8
Townsend Index (M ± SD)	-.7±3.3	-1.3±3.0	-1.5±2.9	-1.5±2.9	-1.2±3.0
Employment status (%)					
In paid employment or self-employed	63.2	60.0	57.4	54.7	53.4
Retired	24.9	31.3	35.3	38.1	38.4
Looking after home and/or family	3.0	3.0	2.7	2.7	2.6
Unable to work because of sickness or disability	5.4	2.9	2.6	2.6	3.4
Unemployed	2.7	1.7	1.2	1.2	1.4
Doing unpaid or voluntary work	0.4	0.5	0.5	0.5	0.5
Full-time or part-time student	0.4	0.2	0.2	0.2	0.3
Qualifications (%)					
College or University Degree	35.0	39.1	40.1	41.2	42.4
A levels/AS levels or equivalent	14.0	14.0	13.7	13.5	12.8
O levels/GCSEs or equivalent	27.5	26.4	26.1	25.4	23.8
CSEs or equivalent	8.9	6.7	6.0	5.7	5.8
NVQ or HND or HNC or equivalent	8.9	7.8	7.5	7.7	8.4
Other professional qualifications	5.7	6.0	6.6	6.6	6.8
Total household income (%)					
Less than 18,000	24.6	21.0	21.1	22.3	25.3
18,000-30,999	23.7	24.5	25.8	26.4	26.7
31,000-51,999	25.2	26.6	26.9	26.4	25.0
52,000-100,000	20.5	21.9	20.6	20.0	18.4
Greater than 100,000	5.9	6.1	5.5	4.9	4.6

Shift work (%)					
Never/rarely	80.4	83.4	84.3	84.1	81.5
Sometimes	8.2	7.2	6.9	6.9	7.9
Usually	2.5	2.0	2.0	2.0	2.3
Always	8.9	7.4	6.9	7.0	8.3
Night shift work (%)					
Never/rarely	47.5	49.9	50.1	51.2	48.0
Sometimes	29.1	28.0	28.3	28.1	29.3
Usually	8.4	7.6	7.9	7.8	7.7
Always	15.1	14.6	13.6	12.9	15.0

Table S3. Baseline characteristics according to milk intake estimations

	**Milk Intake Estimation (Quintiles)**				
	**Low**	**Low/medium**	**Medium**	**Medium/high**	**High**
Number of participants	96543	100710	94754	95455	96896
Mean ± SD	105.15±36.63	181.58±15.71	228.75±12.69	276.25±15.40	385.02±112.08
Sex					
Female (%)	56.3	55.2	54.5	54.4	51.2
Male (%)	43.7	44.7	45.5	45.6	48.8
Age (M ± SD)	54.8±8.3	56.2±8.2	56.9±7.9	57.5±7.8	57.2±7.8
BMI (kg/m2)	27.7±5.1	27.4±4.8	27.3±4.6	27.2±4.5	27.3±4.6
Townsend Index (M ± SD)	-.7±3.3	-1.3±3.0	-1.5±2.9	-1.6±2.8	-1.4±3.0
Employment status (%)					
In paid employment or self-employed	62.1	59.1	57.1	55.2	55.2
Retired	26.9	32.8	35.5	37.9	35.9
Looking after home and/or family	3.6	2.9	2.5	2.5	2.5
Unable to work because of sickness or disability	4.0	2.9	2.8	2.6	4.3
Unemployed	2.4	1.6	1.4	1.2	1.6
Doing unpaid or voluntary work	0.5	0.5	0.4	0.4	0.5
Full-time or part-time student	0.4	0.2	0.2	0.2	0.2
Qualifications (%)					
College or University Degree	41.1	40.0	39.2	39.2	37.2
A levels/AS levels or equivalent	14.1	13.7	13.8	13.2	13.1
O levels/GCSEs or equivalent	25.1	25.7	26.2	26.5	26.2
CSEs or equivalent	6.9	6.7	6.5	6.3	7.0
NVQ or HND or HNC or equivalent	7.2	7.6	8.0	8.2	9.4
Other professional qualifications	5.6	6.2	6.3	6.3	7.1
Total household income (%)					

Less than 18,000	22.7	21.4	21.4	22.5	26.2
18,000-30,999	24.1	24.8	25.7	26.6	26.4
31,000-51,999	25.8	26.5	26.7	26.3	25.1
52,000-100,000	21.1	21.4	20.7	19.8	18.3
Greater than 100,000	6.3	6.0	5.5	4.8	4.1
Shift work (%)					
Never/rarely	82.1	83.8	84.1	83.4	80.1
Sometimes	7.7	7.3	7.0	7.1	7.8
Usually	2.3	2.0	1.8	2.1	2.5
Always	7.8	6.9	7.0	7.5	9.7
Night shift work (%)					
Never/rarely	49.8	50.4	50.5	48.9	46.6
Sometimes	28.4	28.9	28.1	29.2	28.4
Usually	7.8	7.6	7.5	7.9	8.6
Always	14.0	13.1	13.9	14.0	16.4

Table S4. Baseline characteristics according to healthy sleep scores

	**Healthy Sleep Scores**					
	**0**	**1**	**2**	**3**	**4**	**5**
Number of participants	10679	51136	129355	174265	111954	25105
Sex						
Female (%)	46.9	48.5	52.1	55.7	58.3	55.3
Male (%)	53.1	51.5	47.9	44.3	41.7	44.7
Age (M ± SD)	56.6±8.0	57.3±7.8	57.0±7.9	56.5±8.0	55.9±8.2	54.5±8.5
BMI (kg/m2)	30.0±5.9	28.9±5.3	27.9±4.9	27.1±4.5	26.5±4.3	26.0±4.1
Townsend Index (M ± SD)	.1±3.6	-.6±3.3	-1.1±3.1	-1.4±3.0	-1.6±2.8	-1.6±2.8
Employment status (%)						
In paid employment or self-employed	44.2	49.9	55.0	58.7	62.1	67.8
Retired	33.8	36.5	35.5	33.8	31.5	26.1
Looking after home and/or family	2.7	2.5	2.6	2.8	3.0	3.1
Unable to work because of sickness or disability	15.2	8.0	4.2	2.4	1.3	1.0
Unemployed	3.3	2.3	2.0	1.5	1.3	1.1
Doing unpaid or voluntary work	0.4	0.5	0.4	0.5	0.5	0.5
Full-time or part-time student	0.3	0.3	0.2	0.3	0.3	0.3
Qualifications (%)						
College or University Degree	32.9	35.1	37.0	.9.8	42.8	45.8
A levels/AS levels or equivalent	12.6	13.2	13.3	13.8	13.8	13.8
O levels/GCSEs or equivalent	26.9	27.3	27.1	25.9	24.6	22.3

CSEs or equivalent	9.9	7.7	7.1	6.4	5.9	5.8
NVQ or HND or HNC or equivalent	11.0	9.8	8.8	7.8	7.0	6.7
Other professional qualifications	6.7	6.8	6.6	6.4	6.0	5.6
Total household income (%)						
Less than 18,000	40.1	32.6	25.9	21.4	17.9	15.5
18,000-30,999	25.4	26.4	26.6	25.5	24.3	22.2
31,000-51,999	20.2	22.8	25.2	26.7	27.5	27.6
52,000-100,000	11.9	14.9	18.0	20.9	23.5	26.1
Greater than 100,000	2.5	3.4	4.3	5.5	6.8	8.6
Shift work (%)						
Never/rarely	68.8	74.9	80.1	84.0	86.2	86.6
Sometimes	10.3	9.6	8.2	7.1	6.4	6.6
Usually	3.8	2.9	2.5	2.0	1.7	1.6
Always	17.0	12.5	9.3	7.0	5.6	5.1
Night shift work (%)						
Never/rarely	36.1	42.5	47.1	51.3	53.6	54.9
Sometimes	28.5	28.9	28.6	28.4	28.6	29.4
Usually	11.5	9.3	8.4	7.4	6.9	6.8
Always	23.9	19.3	16.0	12.9	10.9	8.8

Table S5. Baseline characteristics according to total number of mental health symptomatology reported

	**Mental Healthy Symptomatology**						
	**0**	**1**	**2**	**3**	**4**	**5**	**6**
Number of participants	51953	61684	57334	55920	52480	48114	42758
Sex							
Female (%)	42.4	44.3	50.6	54.3	56.7	58.5	59.9
Male (%)	57.6	55.7	49.4	45.7	43.3	41.5	40.1
Age (M ± SD)	57.9±7.8	57.3±8.0	57.2±8.0	57.0±8.0	56.6±8.0	56.4±8.1	56.0±8.1
BMI (kg/m2)	27.3±4.4	27.4±4.5	27.3±4.6	27.3±4.6	27.3±4.7	27.3±4.8	27.5±4.9
Townsend Index (M ± SD)	-1.5±2.9	-1.5±2.9	-1.4±2.9	-1.4±2.9	-1.3±3.0	-1.3±3.0	-1.2±3.1
Employment status (%)							
In paid employment or self-employed	55.4	58.3	57.8	57.7	58.2	58.7	59.0
Retired	39.2	36.0	36.0	35.6	34.2	33.0	31.9
Looking after home and/or family	2.0	2.0	2.2	2.5	2.8	2.9	3.2
Unable to work because of sickness or	1.5	1.7	1.9	2.1	2.5	2.9	3.5
disability	1.2	1.4	1.4	1.5	1.6	1.6	1.7
Unemployed	0.4	0.4	0.5	0.4	0.5	0.5	0.4

Doing unpaid or voluntary work	0.2	0.3	0.3	0.2	0.2	0.3	0.3
Full-time or part-time student							
Qualifications (%)							
College or University Degree	41.7	43.4	41.5	40.8	39.5	38.4	37.6
A levels/AS levels or equivalent	13.5	13.1	13.5	13.3	13.6	13.7	13.8
O levels/GCSEs or equivalent	24.7	23.4	24.9	25.7	26.0	26.5	26.9
CSEs or equivalent	5.2	5.2	5.6	5.9	6.6	7.0	7.3
NVQ or HND or HNC or equivalent	8.0	8.3	7.9	7.7	7.8	7.9	8.1
Other professional qualifications	6.9	6.6	6.6	6.5	6.4	6.5	6.3
Total household income (%)							
Less than 18,000	19.8	19.0	20.2	21.3	21.9	23.0	23.8
18,000-30,999	26.0	24.9	25.5	25.5	25.6	25.6	25.5
31,000-51,999	26.7	26.4	26.6	26.2	26.3	26.1	26.4
52,000-100,000	21.3	22.6	21.6	21.2	20.6	20.2	19.8
Greater than 100,000	6.1	7.2	6.2	5.9	5.6	5.1	4.6
Shift work (%)							
Never/rarely	84.4	83.6	83.7	83.3	82.6	82.6	82.0
Sometimes	6.7	7.2	7.0	7.1	7.3	7.5	7.7
Usually	1.9	2.1	2.1	2.0	2.1	2.2	2.1
Always	7.0	7.1	7.2	7.6	7.9	7.7	8.2
Night shift work (%)							
Never/rarely	46.4	47.1	48.3	48.8	49.4	49.6	50.0
Sometimes	30.1	30.5	28.1	29.3	29.1	28.9	27.1
Usually	8.4	8.5	8.5	7.8	7.5	7.6	8.0
Always	15.1	13.9	15.1	14.1	14.0	13.6	15.0

Table S5. Cont.

Baseline characteristics according to total number of mental health symptomatology reported

	**Mental Healthy Symptomatology**						
	**7**	**8**	**9**	**10**	**11**	**12**	**13**
Number of participants	36606	29945	23127	17601	12765	8814	2141
Sex							
Female (%)	61.4	61.5	62.1	62.6	62.0	61.6	47.4
Male (%)	38.6	38.5	37.9	37.4	38.0	38.4	52.6
Age (M ± SD)	55.7±8.0	55.4±8.1	55.2±8.0	55.0±8.0	54.8±7.9	54.2±7.8	53.3±7.9
BMI (kg/m2)	27.4±4.9	27.5±5.0	27.5±5.1	27.5±5.1	27.5±5.2	27.7±5.4	28.0±5.2
Townsend Index (M ± SD)	-1.1±3.1	-1.0±3.1	-.9±3.2	-.8±3.2	-.6±3.3	-.4±3.4	.2±3.6
Employment status (%)							

In paid employment or self-employed	59.0	59.3	58.4	57.2	54.5	52.3	49.9
Retired	30.9	29.4	28.6	28.5	27.4	25.1	20.8
Looking after home and/or family	3.3	3.5	3.6	3.9	4.2	4.7	3.2
Unable to work because of sickness or	4.2	4.9	6.2	7.3	10.3	13.8	19.8
disability	1.8	2.0	2.3	2.2	2.6	3.2	4.7
Unemployed	0.5	0.5	0.6	0.6	0.6	0.6	0.8
Doing unpaid or voluntary work	0.3	0.4	0.3	0.3	0.3	0.3	0.8
Full-time or part-time student							
Qualifications (%)							
College or University Degree	37.3	37.0	36.5	35.7	35.6	34.4	37.2
A levels/AS levels or equivalent	13.5	14.1	14.0	13.7	14.5	14.4	13.6
O levels/GCSEs or equivalent	27.4	27.0	27.1	28.4	26.9	27.8	24.1
CSEs or equivalent	7.6	7.9	8.3	8.6	9.0	9.5	9.4
NVQ or HND or HNC or equivalent	7.9	8.1	8.5	8.2	8.4	8.7	10.4
Other professional qualifications	6.2	5.9	5.5	5.3	5.6	5.2	5.3
Total household income (%)							
Less than 18,000	24.8	26.1	27.4	29.1	31.6	35.4	41.7
18,000-30,999	25.0	25.5	25.4	25.4	25.5	25.6	21.1
31,000-51,999	25.9	25.6	25.2	25.3	23.8	22.0	20.0
52,000-100,000	19.8	18.2	18.3	16.7	16.0	14.2	14.2
Greater than 100,000	4.6	4.6	3.6	3.6	3.1	2.8	3.0
Shift work (%)							
Never/rarely	82.3	81.5	81.3	81.2	80.4	79.5	71.6
Sometimes	7.7	7.9	8.0	8.3	7.9	8.9	12.5
Usually	2.2	2.4	2.2	2.1	2.4	2.7	3.7
Always	7.8	8.2	8.6	8.4	9.3	8.9	12.2
Night shift work (%)							
Never/rarely	51.1	49.7	51.4	52.6	51.8	50.1	50.7
Sometimes	27.4	28.3	26.7	27.3	27.1	27.2	28.3
Usually	7.8	8.8	6.7	5.9	7.1	8.1	5.0
Always	13.6	13.2	15.1	14.2	14.0	14.7	15.4

Table S6. Correlations between mental health questions and total number of mental health symptomatology reported

Items	*N*	*r*
Mood swings	501237	.682**
Miserableness	488185	.642**
Irritability	492100	.515**
Sensitivity/hurt feelings	477971	.611**
Fed up feelings	486166	.650**
Nervous feelings	489929	.538**
Worrier/anxious feelings	487563	.631**
Tense	487609	.520**
Worry too long after embarrassment	482661	.585**
Suffer from nerves	480156	.502**
Loneliness/isolation	481867	.465**
Guilty feelings	492622	.554**
Risk taking	487098	.108**

**. Correlation is significant at the 0.01 level (2-tailed).

Table S7. Cronbach's Alpha Scores if total number of mental health symptomatology items deleted

Items	Cronbach's Alpha if Item Deleted
Mood swings	0.786
Miserableness	0.790
Irritability	0.799
Sensitivity/hurt feelings	0.795
Fed up feelings	0.788
Nervous feelings	0.795
Worrier/anxious feelings	0.793
Tense	0.795
Worry too long after embarrassment	0.797
Suffer from nerves	0.798
Loneliness/isolation	0.801
Guilty feelings	0.796
Risk taking	0.834
