# Supplementary material for: Diet, Sleep, and Mental Health: Insights from the UK Biobank Study
Source: Nutrients. 2021 Jul 27;13(8):2573. doi: 10.3390/nu13082573 (PMC8398967; doi:10.3390/nu13082573)
Supplement: Supplementary file 1 [file nutrients-13-02573-s001.zip › supp methods - revised.pdf]

Supplementary methods

Assessment of diet

In order to assess diet, we used UK Biobank (UKB) Food Frequency Questionnaire scores at baseline. For the current paper, we were interested in (1) UKB cooked vegetable intake (Field ID:1289; On average how many heaped tablespoons of cooked vegetables would you eat per day?), (2) UKB salad/raw vegetable intake (Field ID:1299; On average how many heaped tablespoons of raw vegetables would you eat per day?), (3) UKB fresh fruit intake (Field ID:1309; About how many pieces of fresh fruit would you eat per day?), (4) UKB dried fruit intake (Field ID:1319; About how many pieces of dried fruit would you eat per day?), (5) UKB oily fish intake (Field ID:1329; How often do you eat oily fish? (e.g. sardines, salmon, mackerel, herring); coded as never, less than once a week, once a week, 2-4 times a week, 5-6 times a week, once or more daily, do not know, and prefer not to answer), (6) UKB non-oily fish intake (Field ID:1339; How often do you eat other types of fish? (e.g. cod, tinned tuna, haddock); coded as never, less than once a week, once a week, 2-4 times a week, 5-6 times a week, once or more daily, do not know, and prefer not to answer), (7) UKB processed meat intake (Field ID:1349; How often do you eat processed meats (such as bacon, ham, sausages, meat pies, kebabs, burgers, chicken nuggets)?; coded as never, less than once a week, once a week, 2-4 times a week, 5-6 times a week, once or more daily, do not know, and prefer not to answer), (8) UKB poultry intake (Field ID:1359; How often do you eat chicken, turkey or other poultry? (Do not count processed meats); coded as never, less than once a week, once a week, 2-4 times a week, 5-6 times a week, once or more daily, do not know, and prefer not to answer), (9) UKB beef intake (Field ID:1369; How often do you eat beef? (Do not count processed meats); coded as never, less than once a week, once a week, 2-4 times a week, 5-6 times a week, once or more daily, do not know, and prefer not to answer), (10) UKB lamb/mutton intake (Field ID:1379; How often do you eat lamb/mutton? (Do not

count processed meats); coded as never, less than once a week, once a week, 2-4 times a week, 5-6 times a week, once or more daily, do not know, and prefer not to answer), (11) UKB pork intake (Field ID:1389; How often do you eat pork? (Do not count processed meats); coded as never, less than once a week, once a week, 2-4 times a week, 5-6 times a week, once or more daily, do not know, and prefer not to answer), (12) UKB bread intake (Field ID:1438; How many slices of bread do you eat each week?) and type (Field ID:1448; What type of bread do you mainly eat?; coded as white, brown, wholemeal or wholegrain, other type of bread, do not know, prefer not to answer), (13) UKB cereal intake (Field ID:1458; How many bowls of cereal do you eat a week?) and type (Field ID:1468), (14) UKB tea intake (Field ID:1488; How many cups of tea do you drink each day? (Include black and green tea), (15) UKB coffee intake (Field ID:1498; How many cups of coffee do you drink each day? (Include decaffeinated coffee)), and (16) UKB water intake (Field ID:1528; How many glasses of water do you drink each day?) data fields.

Calculation of healthy diet score. This score was a part of on Bradbury et al.'s [1] healthy lifestyle factor score and it was calculated based on consumption of commonly eaten food groups following recommendations on dietary priorities for cardiometabolic health (Fruits: ≥ 3 servings/day, Vegetables: ≥ 3 servings/day, Fish: ≥ 2 servings/week, Processed meats: ≤ 1 serving/week, Unprocessed red meats: ≤ 1.5 servings/week, Whole grains: ≥ 3servings/day, Refined grains: ≤ 1.5 servings/day).

Calculation of partial fibre intake score. As per Bradbury et al. [2], based on standard portion sizes and an approximate non-starch polysaccharide content, a portion size in grams were assigned to fruits, vegetables, bread, and cereals. Then, the frequency of consumption for each food ('less than one' was coded as 0.5) were multiplied by the fibre content and summed in order to calculate a fibre estimation.

Calculation of milk intake score. We used Bradbury et al.'s [3] milk intake estimates to calculate milk intake scores. Questions on type of milk, bowls of breakfast cereal, cups of tea, and cups of coffee, as well as type of milk they mainly use were used. Participants who reported "never/rarely having milk" were excluded. Then, milk intake estimates were calculated for participants who reported consuming 'Full cream', 'Semi-skimmed' or 'Skimmed' by adding up their daily milk consumption assuming that participants added 100 mL of milk to each bowl of breakfast cereal, 35 mL of milk to each cup of tea, and 25 mL of milk to each cup of coffee.

Assessment of sleep

In order to assess sleep, we used UKB's touchscreen questionnaire on sleep at baseline. Data fields of interest were (1) UKB sleep duration (Field ID:1160; About how many hours sleep do you get in every 24 hours? (please include naps)), (2) Chronotype (Field ID: 1180; Do you consider yourself to be?; coded as definitely a morning person, more a morning than evening person, more an evening than a morning person, definitely an evening person, do not know, prefer not to answer), (3) Sleeplessness/insomnia (Field ID: 1200; Do you have trouble falling asleep at night or do you wake up in the middle of the night?; coded as never/rarely, sometimes, usually, prefer not to answer), (4) Snoring (Field ID: 1210; Does your partner or a close relative or friend complain about your snoring?; coded as yes, no, do not know, prefer not to answer), and (5) Daytime dozing / sleeping (Field ID: 1220; How likely are you to doze off or fall asleep during the daytime when you don't mean to? (e.g., when working, reading, or driving)).

Calculation of healthy diet score. We utilised healthy sleep score calculations of Fan and colleagues [4] that is based on five sleep questions specified above. Low-risk sleep factors were (i) having an early chronotype, (ii) sleeping 7–8 h per day, (iii) never having or rarely having insomnia symptoms, (iv) not reporting snoring, and (v) not reporting frequent

daytime sleepiness. Participants received a score of 1 if they were classified as low risk for that factor and scores for all five factors were added to calculate a healthy sleep score, where higher scores represent healthier sleep patterns.

Assessment of mental health

For mental health assessment, we utilised UKB's touchscreen questionnaire on psychological factors and mental health at baseline. Data fields of interest included (1) UKB mood swings (Field ID:1920; Does your mood often go up and down?), (2) UKB miserableness (Field ID:1930; Do you ever feel 'just miserable' for no reason?), (3) UKB irritability (Field ID:1940; Are you an irritable person?), (4) UKB sensitivity / hurt feelings (Field ID:1950; Are your feelings easily hurt ?), (5) UKB fed-up feelings (Field ID:1960; Do you often feel 'fed-up'?), (6) UKB nervous feelings (Field ID:1970; Would you call yourself a nervous person?), (7) UKB worrier / anxious feelings (Field ID:1980; Are you a worrier?), (8) UKB tense / 'highly strung' (Field ID:1990; Would you call yourself tense or 'highly strung'?), (9) UKB worry too long after embarrassment (Field ID:2000; Do you worry too long after an embarrassing experience?), (10) UKB suffer from 'nerves' (Field ID:2010; Do you suffer from 'nerves'?), (11) UKB loneliness, isolation (Field ID:2020;Do you often feel lonely?), (12) UKB guilty feelings (Field ID:2030; Are you often troubled by feelings of guilt?), (13) UKB risk taking (Field ID:2040; Would you describe yourself as someone who takes risks?). Questions to all answers coded as 'yes', 'no', 'do not know', 'prefer not to answer'.

References

1. Lourida, I., et al., *Association of Lifestyle and Genetic Risk With Incidence of Dementia.* JAMA, 2019. **322**(5): p. 430-7.
2. Bradbury, K.E., et al., *Dietary assessment in UK Biobank: an evaluation of the performance of the touchscreen dietary questionnaire.* Journal of Nutritional Science, 2018. **7**: p. e6.
3. Bradbury, K.E., N. Murphy, and T.J. Key, *Diet and colorectal cancer in UK Biobank: a prospective study.* International Journal of Epidemiology, 2019. **49**(1): p. 246-258.
4. Fan, M., et al., *Sleep patterns, genetic susceptibility, and incident cardiovascular disease: a prospective study of 385 292 UK biobank participants.* Eur Heart J, 2020. **41**(11): p. 1182-1189.
